# Dose-dependent and cell type-specific cell death and proliferation following *in vitro* exposure to radial extracorporeal shock waves

**DOI:** 10.1038/srep30637

**Published:** 2016-08-01

**Authors:** Tanja Hochstrasser, Hans-Georg Frank, Christoph Schmitz

**Affiliations:** 1 Extracorporeal Shock Wave Research Unit, Department of Anatomy II, Ludwig-Maximilians-University of Munich, 80336 Munich, Germany

## Abstract

Radial extracorporeal shock wave (rESW) therapy is widely used in musculoskeletal disorders and wound repair. However, the mechanisms of action are still largely unknown. The current study compared the effects of rESWs on two cell types. Human fetal foreskin fibroblasts (HFFF2) and human placental choriocarcinoma cell line JEG-3 were exposed to 0, 100, 200, 500 or 5000 rESWs generated with a Swiss DolorClast device (2.5 bar, 1 Hz). FACS analysis immediately after rESW exposure showed that initially, rESWs rather induced mechanical cell destruction than regulated or programmed cell death. Cell damage was nearly negated by reducing cavitation. Furthermore, cell viability decreased progressively with higher numbers of rESWs. Exposure to rESWs had no impact on growth potential of JEG-3 cells, but dose-dependently increased growth potential of HFFF2 cells. Cultivation of cells that were initially exposed to sham-rESWs in conditioned media increased the growth potential of HFFF2 cells, nevertheless, an even stronger effect was achieved by direct exposure to rESWs. Additionally, cell cycle distribution analysis demonstrated a shift in proportion from G0/G1 to G2/M phase in HFFF2 cells, but not in JEG-3 cells. These data demonstrate that rESWs leads to initial and subsequent dose-dependent and cell type-specific effects *in vitro.*

Extracorporeal shock wave therapy (ESWT) is widely used in the non-invasive treatment of various diseases of the musculoskeletal system including tendinopathies and soft tissue wounds (for review see, e.g. ref. 1, 2, 3, 4). Contrary to what was argued by Frairia and Berta^5^ the underlying mechanisms of action of ESWT are still largely unknown, although several studies addressed the molecular and cellular mechanisms of ESWT on these conditions (e.g., ref. 6, 7, 8).

Two types of extracorporeal shock waves (ESWs) are used in medical therapy, focused extracorporeal shock waves (fESWs) and radial extracorporeal shock waves (rESWs)^4^. Both are single acoustic impulses with an initial high positive peak pressure between 10 and 100 megapascals (MPa) reached in less than one microsecond (μs)^9^. The positive pressure amplitude is followed by a low tensile amplitude of a few microseconds duration that can generate cavitation^1^,^10^,^11^,^12^. They are further characterized by a short life cycle of approximately 10–20 μs and a broad frequency spectrum. Focused ESW differ from rESW in the penetration depth into the tissue, some physical characteristics, and the technique for generating them^1^,^12^,^13^.

Mechanisms mediating the effects of focused ESWT in musculoskeletal disorders were not only investigated in clinical settings, but also in animal models and *in vitro* (representative studies are summarized in Table 1). Corresponding information about rESWT is mostly lacking.

It has been shown that exposure of cells to ESWs *in vitro* can affect cell proliferation, differentiation, gene expression, growth factor production and cytokine release^14^,^15^,^16^,^17^,^18^,^19^. Furthermore, it has been hypothesized that ESWs can induce biochemical changes through mechanotransduction^20^. Again, the molecular and cellular mechanisms of action are to a large extent unknown.

Various fESW studies were performed on functional activities of cell cultures, however, results are controversial^14^,^16^,^17^,^21^,^22^,^23^,^24^. For example, several studies reported an increase of fibroblast viability after exposure to fESWs *in vitro*^22^,^25^,^26^. In contrast, Kaulesar Johannes *et al*.^22^ found no differences in growth potential between fESW-treated and control fibroblasts, and Gambihler and colleagues^21^ even reported a transitory decrease in proliferation and cell disruption of leukemia cells after exposure to fESWs. On the other hand, many authors reported enhanced biological activities of cells after exposure to fESWs, such as cell proliferation^14^,^16^,^17^,^23^,^24^. Hofmann *et al*.^14^ observed a dose-dependent increase in proliferation of primary osteoblasts 24 to 96 hours after exposure to fESWs. Mesenchymal stem cells *in vitro* also showed not only increased proliferation, but also a higher amount of apoptotic cells 24 and 48 hours after exposure to fESW^16^.

Whereas, few studies have yet explored the effects of rESWs on cell cultures, and none investigated rESW-induced effects on human fibroblasts *in vitro*. In human osteoblast MG63 cells rESW led to reduced cell viability immediately after exposure and a higher growth rate after 24 hours^27^.

In general, effects of fESWs and, to a lesser extent, rESWs were studied in various cell types and models using several different ESWT devices with different parameters and different protocols (number of ESWs, energy flux density). We hypothesized that different cell types are differentially influenced by rESWs. We therefore investigated (1) the effects of rESWs on human fetal foreskin fibroblasts (HFFF2), which are of mesenchymal origin and play a role in the assembly and synthesis of extracellular matrix components and (2) the human choriocarcinoma cell line JEG-3, which is of epithelial origin.

## Results

### Morphological appearance after exposure to rESWs

**Figure 1:**
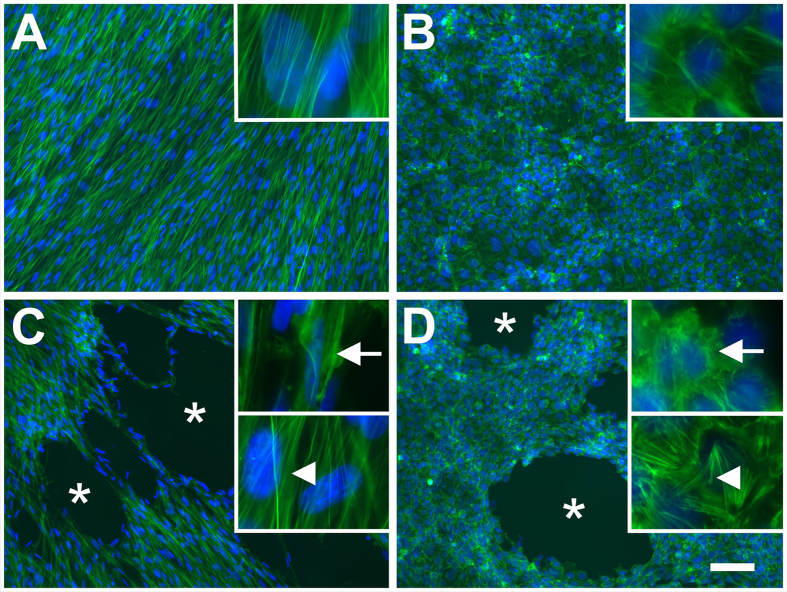
Morphological appearance of adherent cells before and after exposure to radial extracorporeal shock waves. The morphological appearance of HFFF2 (**A**,**C**) and JEG-3 (**B**,**D**) monolayers exposed to sham-rESWs (**A,B**) and rESWs (**C**,**D**) was assessed by immunofluorescence staining with phalloidin (green) and DAPI (4,6-diamidino-2-phenyl-indole (blue). Cells in (**C,D**) were exposed to 100 rESWs as explained in detail in the main text. Cells exposed to sham-rESWs showed a homogeneous cell distribution (**A,B**). Exposure to rESWs caused cellular detachment and, thus, holes in the monolayers (asterisks in **C,D**) as well as disruption of actin filaments in cells located next to the holes (arrows in the upper insets in **C,D**). Cells distant to the holes in the monolayers appeared normal (arrowheads in the lower insets in **C,D**). The scale bar represents 100 μm in the low-power photomicrographs in (**A–D**) and 14 μm in the high-power insets in (**A–D**).

Gross observation of monolayers of HFFF2 and JEG-3 cells exposed to sham-rESWs and stained with phalloidin and DAPI did not show irregularities in the distribution pattern of the cells, whereas exposure to rESWs caused cellular detachment (Fig. 1). The number and size of cell-free areas increased with increasing number of rESWs. Cells in the vicinity of cell-free areas exhibited a ripped appearance, whereas cells in the wider area showed no changes in cell morphology (Fig. 1). Detached cells were found as aggregated cells and ruptured cell debris in the medium.

### Primary effects of rESWs on cell viability

**Figure 2:**
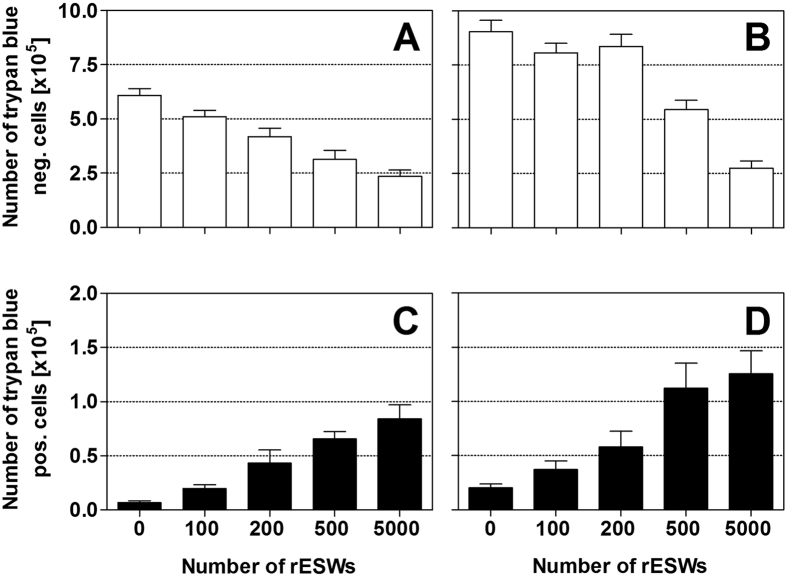
Cell viability after exposure to radial extracorporeal shock waves. Data show absolute numbers (mean ± SEM) of trypan blue negative HFFF2 (**A**) and JEG-3 (**B**) cells as well as of trypan blue positive HFFF2 (**C**) and JEG-3 (**D**) cells as a function of the number of applied rESWs. Results of statistical analysis are summarized in Table 2.

The trypan blue exclusion assay showed that exposure of HFFF2 and JEG-3 cells to rESWs initially reduced the number of trypan blue negative cells compared to cells exposed to sham-rESWs (Fig. 2A,B and Table 2). The number of trypan blue negative cells remained steady with 100 rESWs. With higher numbers of rESWs, the number of trypan blue negative cells decreased progressively. When 500 or 5000 rESWs were applied, cell viability significantly decreased statistically (*p* < 0.001) in both cell lines compared to cells exposed to sham-rESWs (Fig. 2A,B and Table 2).

In line with this, the number of trypan blue positive cells increased after exposure to rESWs in both cell lines compared to cells exposed to sham-rESWs (Fig. 2C,D and Table 2). Exposure to 500 or 5000 rESWs resulted in a statistically significant (*p* < 0.001) increase of the number of trypan blue positive cells (Fig. 2C,D and Table 2).

**Figure 3:**
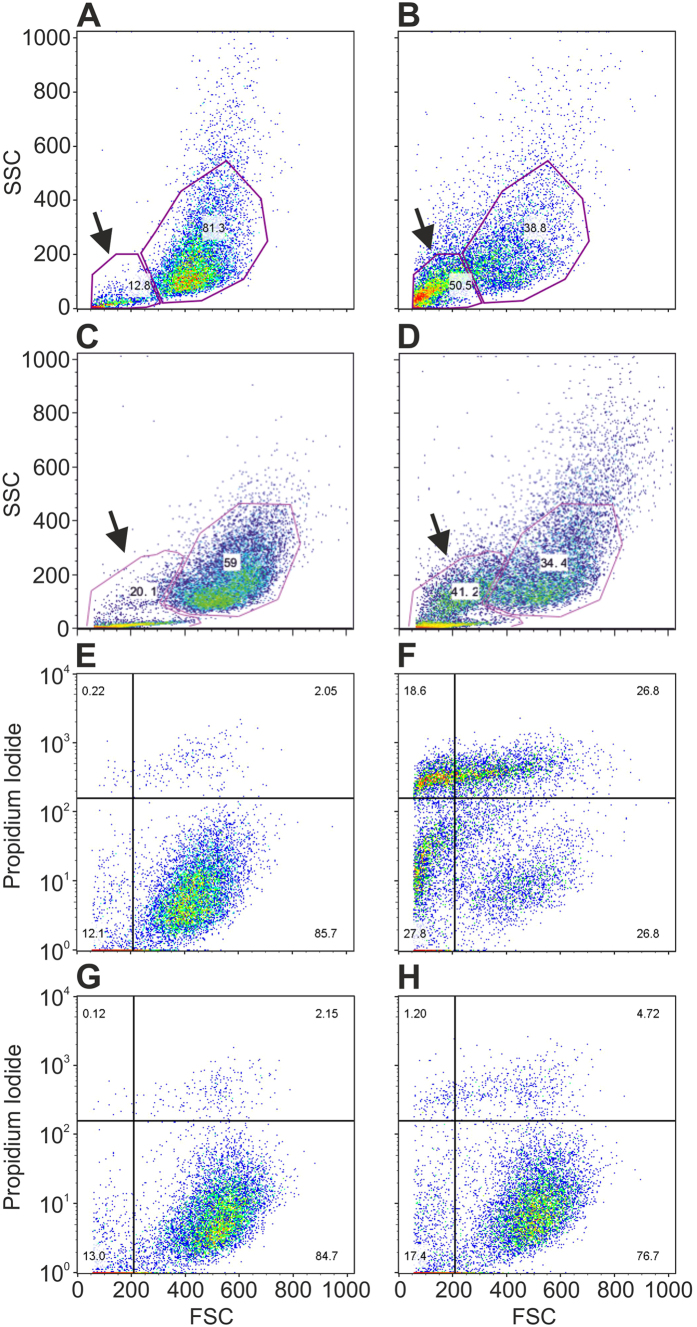
Viable cells and debris/dead cells after exposure to radial extracorporeal shock waves. (**A**–**D**) Original dot-plots of side light scatter (SSC) vs. forward light scatter (FSC) obtained by flow cytometry (FACS Calibur flow cytometer, BD Biosciences, Heidelberg, Germany) of HFFF2 (**A,B**) and JEG-3 (**C,D**) cells after exposure to sham-rESWs (**A,C**) or 500 rESWs (**B,D**). The arrows indicate the fraction of debris/dead cells that was increased between two-fold (JEG-3 cells; **C,D**) and four-fold (HFFF2 cells; **A,B**) immediately after exposure to 500 rESWs compared to exposure to sham-rESWs. (**E–H**), original dot-plots of propidium iodide vs. FSC of HFFF2 cells undergoing cell death after exposure to sham-rESWs (**E,G**) or 500 rESWs (**F,H**) in culture medium (**E,F**) or 10% polyvinyl alcohol solution (**G,H**).

FACS analysis revealed that exposure of HFFF2 and JEG-3 cells to 500 rESWs immediately reduced the relative number of viable cells in comparison to cells exposed to sham-rESWs (Fig. 3A–D), while the relative number of debris/dead cells immediately increased two- to four-fold after exposure to 500 rESWs in both cell lines compared to cells exposed to sham-rESWs (Fig. 3A–D).

In contrast, exposure to 500 rESWs in 10% polyvinyl alcohol (PVA) solution reduced rESW effects on HFFF2 cells. Specifically, fewer cells were propidium iodide-positive after exposure to 500 rESWs in 10% PVA solution and, overall, rESW treatment in 10% PVA solution resulted in a reduced number of debris (Fig. 3E–H).

### Effects of rESW on cell count

**Figure 4:**
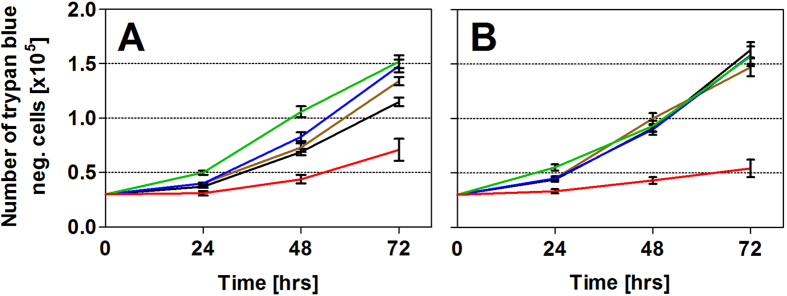
Cell count after exposure to radial extracorporeal shock waves. Data show absolute numbers (mean ± SEM) of HFFF2 (**A**) and JEG-3 (**B**) cells that were trypan blue negative after exposure to 0 (sham-rESWs, black), 100 (brown), 200 (blue), 500 (green) and 5000 (red) rESWs as a function of time after exposure (0, 24, 48 and 72 hours). Results of statistical analysis are summarized in Table 3.

Following a 24 hour period after exposure to rESWs, none of the investigated cell types showed a statistically significant difference in mean numbers of cells between cells exposed to sham-rESWs and those cells that were trypan blue negative after exposure to rESWs (Fig. 4 and Table 3). Compared to cells exposed to sham-rESWs, exposure of HFFF2 cells to 100 rESWs did not result in an effect after 48 hours, but in a statistically significant (*p* < 0.05) increase in the mean number of cells after 72 hours (Fig. 4A and Table 3). Exposure to 200 and 500 rESWs led to a statistically significant (*p* < 0.005) increase in the mean number of HFFF2 cells after both 48 and 72 hours (Fig. 4A and Table 3). In contrast, exposure to 5000 rESWs resulted in a statistically significant (*p* < 0.001) reduction in the mean number of cells (Fig. 4A and Table 3).

At 48 and 72 hours after seeding, JEG-3 cells exposed to 100, 200 and 500 rESWs showed a similar cell count than cells exposed to sham-rESWs, whereas JEG-3 cells exposed to 5000 rESWs showed a statistically significantly (*p* < 0.001) lower number of cells (Fig. 4B and Table 3).

### Effects of conditioned medium on cell count

**Figure 5:**
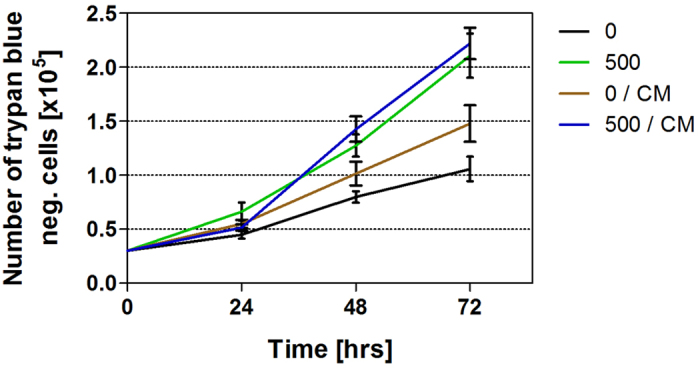
Effect of conditioned medium on cell count. Data show absolute numbers (mean ± SEM) of the following groups of HFFF2 cells that were trypan blue negative as a function of time after exposure (0, 24, 48 and 72 hours): (i) cells exposed to sham-ESW and cultured in fresh culture medium (black); (ii) cells exposed to 500 rESW impulses and cultured in fresh culture medium (green); (iii) cells exposed to sham-ESW and cultured in conditioned medium (brown); and (iv) cells exposed to 500 rESW impulses and cultured in conditioned medium (blue). Results of statistical analysis are summarized in Table 4.

Compared to sham-rESWs, exposure of HFFF2 cells to 500 rESWs and further cultivation either in fresh culture medium or conditioned medium led to a statistically significant increase in the mean number of HFFF2 cells after both 48 and 72 hours, with the effect being slightly stronger in conditioned medium (Fig. 5 and Table 4). Conditioned medium, on its own, did not have any influence on the morphological appearance of sham-rESW cells 48 hours after seeding, but did result in a statistically significant (p < 0.05) increase in the mean number of HFFF2 cells after 72 hours compared to cells exposed to sham-rESWs in fresh culture medium (Fig. 5 and Table 4).

### Effects of rESW on cell cycle

**Figure 6:**
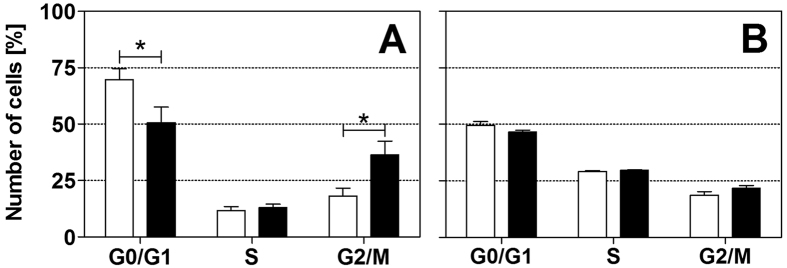
Cell cycle phase distribution after exposure to radial extracorporeal shock waves. Data show relative numbers (mean ± SEM) of HFFF2 (**A**) and JEG-3 (**B**) cells in the G0/G1, S and G2/M phases 24 h after exposure to sham-rESWs (open bars) or to 500 rESWs (closed bars). *p < 0.05.

FACS analysis of cell cycle phase distribution, based on DNA content, revealed statistically significant (*p* < 0.05) differences between HFFF2 cells exposed to rESWs compared to cells exposed to sham-rESWs 24 hours after exposure (Fig. 6A and Supplementary Fig. 1). Specifically, exposure to 500 rESWs reduced the relative number of HFFF2 cells in the G0/G1-phase (G0/G1_sham-rESW_: 69.8 ± 4.8%, G0/G1_500_: 50.6 ± 6.9%; *p* < 0.05) and increased the relative number of HFFF2 cells in the G2/M phase of the cell cycle (G2/M_sham-rESW_: 18.2 ± 3.5%, G2/M_500_: 36.4 ± 6.1%; *p* < 0.05; Fig. 6A). There were no statistically significant changes in the relative number of HFFF2 cells in the S-phase between exposure to sham-rESWs and to rESWs (S_sham-rESW_: 11.7 ± 1.7%, S_500_: 13.1 ± 1.5%; *p* > 0.05; Fig. 6A). In contrast, JEG-3 cells showed similar percentages of the G0/G1, S and G2/M phases in the cell cycle distribution 24 hours after exposure to rESWs compared to sham-rESWs (Fig. 6B and Supplementary Fig. 1).

## Discussion

The effectiveness of ESWT in musculoskeletal conditions has been demonstrated in various studies in the literature^1^,^2^,^4^. With regard to biological effects of ESWT on fibroblasts, Frairia and Berta^5^ even postulated that the physical principles as well as the tissue effects of ESWT have been widely investigated. Unfortunately, this is not the case and many questions about molecular and cellular mechanisms of action, particularly in the field of rESWT, have remained unanswered.

The present *in vitro* study revealed, for the first time, cell-type specific effects of rESWs on human fetal foreskin fibroblasts (HFFF2). In order to determine whether these effects are cell type-specific, experiments were also performed on the human placental choriocarcinoma cell line JEG-3 as control. Both types of cells grow adherently.

The initial effect of rESWs on HFFF2 and JEG-3 cells was progressively increased cellular damage, shown by decreased ability of the cells to exclude trypan blue. Immediate cellular damage of HFFF2 and JEG-3 cells after exposure to rESW in culture medium was also shown by FACS analysis. This result is in agreement with previous fESW and rESW studies in the literature^21^,^28^,^29^,^30^,^31^. Smits *et al*.^28^ compared fESW effects on different types of tumor cells in two treatment models, i.e., single cell suspension vs. cell pellet. In both cases, a dose dependent direct cytotoxicity, established by trypan blue exclusion assay, was observed after exposure to 1000 or 2000 fESWs (energy flux density [EFD] not specified by the authors). The different cell lines showed a different susceptibility, which goes in line with the results of the present study. In addition, Hausdorf *et al*.^26^ reported decreased cell viability of human fibroblasts immediately after exposure to 250 and 500 fESWs (generated with an electrohydraulic fESW source at 25 kV and 3 Hz; EFD not speficied by the authors). Berta *et al*.^25^ even observed a constant decrease of fibroblast viability in relation to the number of fESWs (EFD = 0.05 to 1.48 mJ/mm^2^). In the case of rESWs, Murata *et al*.^30^ showed that cell viability of rabbit chondrocytes sharply decreased to 47% after exposure to 200 rESWs and dramatically to 6.2% after exposure to 5000 rESWs (Swiss DolorClast, 3 bar air pressure, 10 Hz). These results, in accordance with the literature, imply that initially rESWs rather induced mechanical cell damage than regulated or programmed cell death, where typically earliest signs are seen 1–2 hours following exposure^32^. Furthermore, it could be shown in the present study that cell death and destruction of HFFF2 cells by rESWs is predominantly a cavitation-mediated effect. In this context, Angstman *et al*.^11^ showed that rESW exposure of *Caenorhabditis elegans* in polyvinyl-alcohol solution (which is known to reduce cavitation^33^) resulted in reduced locomotion of the worms, implicating primary blast effects as damaging components^11^,^12^. A mechanical mechanism of rESWs on soft tissue was also suspected by Waugh *et al*.^8^. These authors investigated the real-time biological response of healthy and pathological tendons of humans to rESWs (Swiss DolorClast, 2500 impulses administered at 8 Hz, total energy delivered 160 mJ/mm^2^) using microdialysis. The results obtained by Waugh *et al*.^8^ suggest that the mechanical stimulus provided by rESWT might play a role in the initiation of tendon regeneration by promoting pro-inflammatory and catabolic processes that are associated with removing damaged matrix constituents. It is of note that Waugh *et al*.^8^ did not find statistically significant differences between the biological tissue response to rESWs in healthy and pathological tendons. Han *et al*.^19^ obtained contradictory results by treating tendinopathy-affected tenocytes with fESWs (EFD = 0.17 mJ/mm^2^). These authors found statistically significant differences between cell responses in diseased and healthy human tendon cells. They further reported a significant decrease in certain matrix metalloproteinases (MMPs) and interleukins (ILs) (including MMP1, MMP13 and IL6) after exposure to fESWs and speculated about an adverse effect of fESWT on cells as the mechanism of action.

It is currently unknown whether initial cell death (as shown in the present study *in vitro*) also occurs in patients after rESWT *in vivo*, and it remains to be addressed whether this may trigger a beneficial inflammatory response in the tissue healing mechanism, which was hypothesized by Waugh *et al*.^8^, or whether ESWT may prevent a harmful inflammatory response, which was hypothesized by Han *et al*.^19^.

In a model of subcutaneous xenograft implantation in mice, higher numbers of macrophages and increased tumor necrosis factor alpha and interleukin-6 mRNA expression levels were found after exposure to fESWs (EFD = 0.1 mJ/mm^2^)^34^. Furthermore, these authors found higher protein levels of the pivotal macrophage recruitment factors MIF (macrophage migration inhibitory factor) and MIP-1β (macrophage inflammatory protein 1 beta) that could be responsible for the increased macrophage recruitment after exposure to fESWs^34^. Macrophages are essential for wound healing, and they secrete a number of factors such as cytokines and growth factors^35^,^36^, which attract additional cells involved in the proliferative process of wound healing.

To quantify cell growth of HFFF2 and JEG-3 cells, a daily cell count was performed in the present study. This cell count revealed that after 24 hours, the number of cells exposed to rESWs was not significantly modified statistically for both cell types, whereas exposure to rESWs resulted in cell type-specific and number-of-rESWs-dependent alterations after 48 and 72 hours.

Many studies showed that fESWs can induce cell proliferation^14^,^16^,^17^,^23^,^24^. An increase in the proliferation rate of mesenchymal stem cells exposed to fESWs was shown after 24 and 48 hours^16^ (EFD not specified by these authors). Cultured fibroblasts and tenocytes showed an increase in proliferation at long time points (i.e., after 4 to 12 days following exposure to fESWs; EFD = 0.14 or 0.22 mJ/mm^2^), which was explained by a delayed increase in the proliferative activity of cells that survived exposure to fESW^23^,^25^. Moreover, exposure of human bone marrow stromal cells to fESWs (EFD = 0.2 mJ/mm^2^) led to an initial increase of proliferation after six hours that only lasted 12 hours post fESW exposure^25^. In contrast, Gambihler *et al*.^21^ reported a reduced growth potential of L1210 mouse leukemia cells during the first 24 hours after exposure to fESWs (EFD not specified by these authors), while after this period cells continued to proliferate at the same rate as sham-treated cells. Corresponding *in vitro* data have not been published for rESWs.

It is of note that cultivating HFFF2 cells exposed to sham-rESWs in conditioned media from rESW-treated cells resulted in increased cell growth, indicating an indirect effect through mediator release by rESW-damaged cells during exposure to rESWs. At the same time, cell growth of HFFF2 cells was strongly attributed to a direct effect of mechanical stimulation, since cells exposed to rESWs showed enhanced cell growth independent from initial mediator release.

The literature and the present study indicate that exposure of cells to ESWs *in vitro* has a cell type-specific effect on cell number and proliferation activity. It is likely that exposure of rapidly growing cancer cells (such as JEG-3 and L1210 cells^21^) to ESWs does not result in any additional growth and perhaps even enhances susceptibility and growth inhibition. On the other hand, exposure of moderately growing cell types (such as fibroblasts) to ESWs may enhance the proliferation rate of these cell types. In accordance with this hypothesis, cell cycle analysis demonstrated a statistically significant shift in the proportion of cells in G0/G1 phase to G2/M phase in HFFF2 cells exposed to rESWs in the present study. In contrast, the cancer cell line investigated in the present study (i.e., JEG-3 cells) showed no alterations in the cell cycle phase distribution after exposure to rESWs.

The main limitation of the present study is that cells and not whole tissue were used. Cells *in vitro* are being studied in the absence of their local environment that includes interactions with different cell types. Therefore, the optimal approach would be the treatment and analysis of “live” tissue, which would present a very interesting future experiment. However, the use of cell lines offers several advantages over *in vivo* experiments including a pure cell population that provides the basis for reproducible results and a basic understanding of general rESW-induced mechanisms.

In conclusion, the results of the present study indicate that exposure of cells to rESWs *in vitro* initially and subsequently leads to dose-dependent and cell type-specific effects. Radial ESWs did not influence mean numbers of JEG-3 cells, but dose-dependently increased mean numbers of HFFF2 cells. This cell type-specific action of rESWs should be considered depending on the purpose of clinical application of rESWs. These findings further suggest that rESWs work through two different mechanisms, the first involving a less intense indirect effect through mediators released by rESW-damaged cells and a second involving a strong direct mechanical mechanism by rESWs itself. Ultimately, both lead to biological alterations that may trigger tissue healing mechanisms. Further studies will address the question as to whether a direct mechanical and/or an initial destructive effect is one of the pivotal “biological mechanisms” to shock wave treatment as specified by Wang^7^.

## Materials and Methods

### Cell cultures

Human fetal foreskin fibroblasts (HFFF2; obtained from Sigma-Aldrich, Taufkirchen, Germany; Catalog-No. 86031405) were propagated in Dulbecco’s minimum essential medium with high glucose supplemented with 10% fetal bovine serum (FBS) and 1% penicillin/streptomycin (all from Gibco, Life Technologies GmbH, Darmstadt, Germany). HFFF2 cells from passage 11 (P11) were used for the experiments. Human placental choriocarcinoma cell line JEG-3 (DSMZ-German Collection of Microorganisms and Cell Cultures, Braunschweig, Germany; DSMZ-No. ACC-463) was cultured in Ham’s F12 nutrient mixture supplemented with 10% FBS and 1% gentamicin (all from Gibco). Cells in 75 cm^2^ culture flasks (Carl Roth, Karlsruhe, Germany) were incubated at 37 °C in a humidified 95% air, 5% CO_2_ atmosphere.

### Exposure to radial extracorporeal shock waves

Cells (6 × 10^5^ cells/well) were seeded into six-well culture plates (VWR, Ismaning, Germany) and were incubated at 37 °C, 5% CO_2_ for another 24 hours. The cells were exposed to 100, 200, 500 or 5000 rESWs using the handpiece of a radial extracorporeal shock wave device, Swiss DolorClast (Electro Medical Systems, Nyon, Switzerland) equipped with a 36-mm applicator. The handpiece was set vertically in a drill stand (Wolfcraft, Kempenich, Germany). The applicator tip was lowered into the surface of the liquid medium and fixed in this position. The air pressure of the device was set to 2.5 bar and the application frequency to 1 Hz (EFD = 0.10 mJ/mm^2^). During application of rESWs, the cells were outside of the incubator at room temperature. The control group (sham-rESWs) was not exposed to rESWs, but was maintained outside of the incubator with the device off for the same period of time, ranging from 3 to 83 minutes.

### Assessment of cell viability and cell count

After exposure to rESWs or sham-rESWs, cells were immediately counted using a hemocytometer, and cell viability was determined by a 0.4% trypan blue (Sigma-Aldrich) exclusion assay^37^. To assess cell viability and cell count, cells exposed to rESW and sham-rESW were reseeded in triplicates into 24-well plates (Greiner Bio-One GmbH, Frickenhausen, Germany) to continue cultivation. Cell numbers were adjusted to 3 × 10^4^ trypan blue negative cells/well, as determined by trypan blue (Sigma-Aldrich) exclusion assay. After 24, 48 and 72 hours rESW cells and sham-rESW cells were counted. Cell viability was again determined by a 0.4% trypan blue (Sigma-Aldrich) exclusion assay.

To assess the influence of conditioned medium on cell count, HFFF2 cells were exposed to 500 rESWs or sham-rESWs. HFFF2 cells were counted and 3 × 10^4^ trypan blue negative cells/well were reseeded either in fresh culture medium or conditioned medium, which originated from the rESW exposure (500 impulses). After 24, 48 and 72 hours cells exposed to rESWs as well as cells exposed to sham-rESWs in fresh culture medium or conditioned medium were counted with 0.4% trypan blue (Sigma-Aldrich).

To determine the mechanism of cell death, HFFF2 cells were exposed to 500 rESWs or to sham-rESWs either in liquid medium or 10% PVA (31,000 g/mol; Mowiol 4–88, Karl Roth, Karlsruhe, Germany) solution. Immediately after exposure to rESWs or sham-rESWs, cells were harvested and washed with PBS. Propidium iodide (PI) staining solution (50 μg/ml; Sigma-Aldrich) was added for 1 minute and 20,000 cells each were collected with a FACS Calibur flow cytometer (BD Biosciences, Heidelberg, Germany). Results were analyzed using FlowJo Single Cell Analysis Software (FlowJo, Ashland, OR, USA). All cells were identified using side light scatter (SSC) vs. forward light scatter (FSC). SSC/FCS characteristics and PI staining were used to gate viable cells and debris/dead cells.

### Flow cytometric analysis of cell cycle

Cells exposed to 500 rESWs or sham-rESWs were further incubated at 37 °C in humidified 95% air, 5% CO_2_ atmosphere. After 24 hours, cells were harvested and fixed in ice-cold 70% methanol (Merck Millipore, Darmstadt, Germany) at 4 °C for one hour. Cells were washed with PBS containing 2% FBS (Gibco), resuspended in PBS and treated with ribonuclease A (100 μg/ml; Sigma-Aldrich) at 37 °C for 30 minutes. Propidium iodide (PI) staining solution (Sigma-Aldrich) was added and 80,000 cells each were collected with a FACS Calibur flow cytometer (BD Biosciences). A cell cycle histogram, based on DNA contents of PI-positive nuclei, was automatically generated for each sample using CellQuest Pro software (BD Biosciences). Propidium iodide-containing cells were assigned to the G0/G1, S, or G2/M phases by manually drawing gates (Supplementary Fig. 1). The percentage of PI-containing cells in each gate represented the relative number of cells in G0/G1, S, and G2/M phases.

### Immunofluorescence staining

To visualize F-actin, cells exposed to rESWs or sham-rESWs were fixed in 4% phosphate buffered formaldehyde (Roti-Histofix, Carl Roth) for 10 minutes, permeabilized with 0.5% Triton X-100 (Merck Millipore) for 5 minutes, blocked with 5% milk containing 0.2% Triton-X100 (Merck Millipore) for 1 hour at 37 °C, and incubated with phalloidin/Alexa Fluor 488 (Life Technologies) for 20 minutes. Cell nuclei were counterstained using DAPI (4,6-diamidino-2-phenyl-indole; Life Technologies).

### Microscopy

Microscopic images were acquired with a Zeiss AxioCam HRc digital camera (4164 × 3120 pixels; Carl Zeiss MicroImaging, Jena, Germany) attached to a Zeiss Axiophot microscope (Zeiss) and AxioVision software (version 4.8.2; Zeiss) using a 10x and 100x objective. The final figures were assembled using Corel Photo-Paint X6 and Corel Draw X6 (both versions 16.1.0.843; Corel, Ottawa, Canada). Only minor adjustments of contrast and brightness were made, without altering the appearance of the original images.

### Statistical analysis

Primary effects of rESW on cell viability were analyzed using one-way ANOVA followed by Bonferroni *post hoc* tests for pairwise comparisons. Cell count and cell cycle assays were tested by two-way ANOVA followed by Bonferroni *post hoc* tests. Calculations were performed using GraphPad Prism (version 5.04 for Windows; GraphPad Software Inc., San Diego, CA, USA).

All values were expressed as arithmetic means ± standard error of the mean (SEM) from at least three independent experiments. Furthermore, each experiment was based on measurements in triplicates. P values < 0.05 were considered statistically significant.

## Additional Information

**How to cite this article**: Hochstrasser, T. *et al*. Dose-dependent and cell type-specific cell death and proliferation following *in vitro* exposure to radial extracorporeal shock waves. *Sci. Rep.* **6**, 30637; doi: 10.1038/srep30637 (2016).

## Supplementary Material

Supplementary Information

## Figures and Tables

**Table 1:** Examples of studies investigating the effects of focused (fESWs) and radial (rESWs) extracorporeal shock waves on musculoskeletal disorders including tendons in clinical settings, animal models, and fibroblast cultures.

	fESWs	rESWs	
Clinical setting	Reviewed in Schmitz *et al*.^4^	Reviewed in Schmitz *et al*.^4^	
Animal models	Reviewed in Visco *et al*.^38^	—	
Fibroblast culture			
Viability	Kaulesar Johannes *et al*.^22^Berta *et al*.^25^Hausdorf *et al*.^26^	—	
Growth potential	Kaulesar Johannes *et al*.^22^Berta *et al*.^25^	—	
Gene expression	Berta *et al*.^25^ (TFG-β1,collagen type I and III)	—	
Growth factor release	Hausdorf *et al*.^26^ (FGF-2, TGF-β1)	—	
Cell cycle changes	—	—	

**Table 2:** Details of the statistical analysis of the quantitative analysis of cell viability after exposure to radial extracorporeal shock waves.

Cell type	P (ANOVA)	P (Bonferroni *post hoc* tests for pairwise comparisons)									
		0 vs 100	0 vs 200	0 vs 500	0 vs 5000	100 vs 200	100 vs 500	100 vs 5000	200 vs 500	200 vs 5000	500 vs 5000
Trypan blue negative cells											
HFFF2	***	ns	***	***	***	ns	**	***	ns	*	ns
JEG-3	***	ns	ns	***	***	ns	*	***	*	***	*
Trypan blue positive cells											
HFFF2	***	ns	**	***	***	ns	***	***	ns	**	ns
JEG-3	***	ns	ns	***	***	ns	**	***	ns	**	ns

Primary effects of rESWs on cell viability were analyzed using one-way ANOVA followed by Bonferroni *post hoc* tests for pairwise comparisons. *p < 0.05; **p < 0.01; ***p < 0.001; ns, not significant.

**Table 3:** Details of the statistical analysis of the quantitative analysis of cell count.

Cell type	P (ANOVA)			P (Bonferroni *post hoc* tests for pairwise comparisons)				
	Interaction	Number of rESWs	Time		0 vs 100	0 vs 200	0 vs 500	0 vs 5000
HFFF2	***	***	***	48 hrs	ns	*	***	***
				72 hrs	*	***	***	***
JEG-3	***	***	***	48 hrs	ns	ns	ns	***
				72 hrs	ns	ns	ns	***

Cell counts were analyzed by two-way ANOVA followed by Bonferoni *post hoc* tests for pairwise comparisons. The table shows results of comparisons between mean numbers of cells exposed to sham-rESWs and of cells exposed to rESWs at 48 and 72 hours after exposure. *p < 0.05; ***p < 0.001; ns, not significant.

**Table 4:** Details of the statistical analysis of the quantitative analysis of cell count influenced by conditioned medium (CM).

Cell type	P (ANOVA)			P (Bonferroni *post hoc* tests for pairwise comparisons)			
	Interaction	Number of rESWs/CM	Time		0 vs 0/CM	0 vs 500	0 vs 500/CM
HFFF2	***	***	***	48 hrs	ns	*	***
				72 hrs	*	***	***

Cell counts were analyzed by two-way ANOVA followed by Bonferoni *post hoc* tests for pairwise comparisons. The table shows results of comparisons between mean numbers of cells exposed to sham-rESWs (cultured either in fresh culture medium or conditioned medium) and of cells exposed to 500 rESWs (cultured either in fresh culture medium or conditioned medium) at 48 and 72 hours after exposure. *p < 0.05; ***p < 0.001; ns, not significant.
