# Supplementary material for: Dose-dependent and cell type-specific cell death and proliferation following in vitro exposure to radial extracorporeal shock waves
Source: Sci Rep. 2016 Aug 1;6:30637. doi: 10.1038/srep30637 (PMC4967921; doi:10.1038/srep30637)
Supplement: Supplementary Information [file srep30637-s1.doc]

**Dose-dependent and cell type-specific cell death and proliferation following**

***in vitro* exposure to radial extracorporeal shock waves**

Tanja Hochstrasser^1^, Hans-Georg Frank^1^, Christoph Schmitz^1^

^1^ Extracorporeal Shock Wave Research Unit, Department of Anatomy II, Ludwig-Maximilians-University of Munich, 80336 Munich, Germany

**Supporting Information**


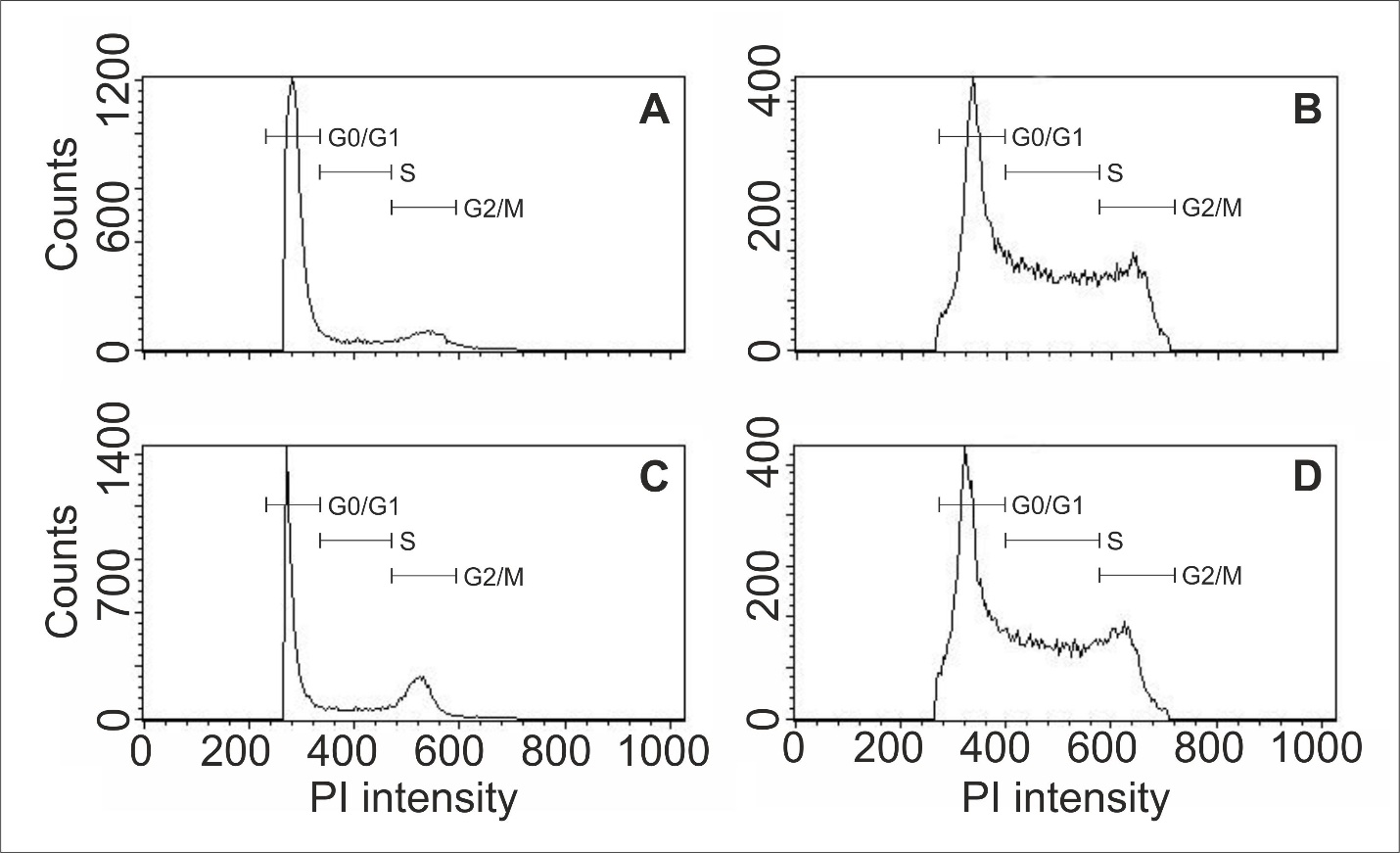


**Supplementary Figure 1. Cell cycle phase distribution** **after exposure to radial extracorporeal shock waves (rESWs).** Data show characteristic DNA histograms of HFFF2 cells exposed to sham-rESWs (A) or rESWs (C), as well as of JEG-3 cells exposed to sham-rESWs (B) or rESW JEG-3 (D), obtained with the FACS Calibur flow cytometer (BD Biosciences, Heidelberg, Germany).
